# Evaluation of neural tube defects from 2014 to 2019 in Turkey

**DOI:** 10.1186/s12884-022-04678-z

**Published:** 2022-04-19

**Authors:** Nilgün Çaylan, Sıddıka Songül Yalçin, Başak Tezel, Şirin Aydin, Oben Üner, Fatih Kara

**Affiliations:** 1 Ministry of Health, General Directorate of Public Health, Ankara, Turkey; 2 Department of Pediatrics, Division of Social Pediatrics, Hacettepe University Faculty of Medicine, 06100 Ankara, Turkey

**Keywords:** Neural tube defect, Prevalence, Mortality, Turkey, Folic acid, Supplementation, Fortification

## Abstract

**Objective:**

The aim of this study is to determine the frequency of neural tube defects (NTDs) and to examine the epidemiological characteristics of NTD related deaths in Turkey.

**Methods:**

This nationwide descriptive study was included NTD related infant deaths, termination of pregnancy for fetal anomaly (ToPFA) and stillbirth cases registered in Death Notification System between 2014 and 2019, and patients diagnosed with NTD in the 2018 birth cohort.

**Findings:**

In the 2018 birth cohort, there were 3475 cases of NTD at birth (27.5 per 10,000). The fatality rates for live-born babies with NTD in this cohort were 13.5% at first year, and 15.6% at the end of March, 2022. NTDs were associated with 11.7% of ToPFA cases, 2.5% of stillbirths and 2.8% of infant deaths in 2014–2019. NTD related stillbirth rate was 1.74 per 10,000 births, while NTD related ToPFA rate and infant mortality rate were 0.61 and 2.70 per 10,000 live births respectively. NTD-related stillbirth and infant mortality rate were highest in the Eastern region (3.64 per 10,000 births; 4.65 per 10,000 live births respectively), while ToPFA rate was highest in the North and West regions (1.17 and 0.79 per 10,000 live births respectively) (*p* < 0.05). Prematurity and low birth weight were the variables with the highest NTD related rates for stillbirths (11.26 and 16.80 per 10,000 birth), ToPFA (9.25 and 12.74) per 10,000 live birth), and infant deaths (13.91 and 20.11 per 10,000 live birth) (*p* < 0.05).

**Conclusion:**

NTDs are common and have an important place among the mortality causes in Turkey. Primary prevention through mandatory folic acid fortification should be considered both to reduce the frequency of NTD and related mortality rates.

## Introduction

Neural tube defects (NTDs) are among the common and serious congenital anomalies that cause hundreds of thousands of deaths, as well as lifelong disability in survivors [1, 2]. According to the March of Dimes estimates, in the year 2001, 323,904 cases of NTD were present globally (25 per 10,000 live births) [3]. A recent meta-analysis study showed that, for 2015, it was estimated that there were 260,100 (uncertainty range (UI): 213800, 322,000) NTD affected birth outcomes worldwide (prevalence 18.6; 15.3, 23.0/10000 live births) [4].

NTDs result from secondary to failure of the neural plate to close during embryonic development (postconceptional 21st to 28th days) [5]. Classically, NTDs are divided into two main groups: defects affecting brain structures (anencephaly and encephalocele) and structures of the spinal cord (meningocele, myelomeningocele, and other forms of spina bifida) [1, 6]. The etiology of NTDs is multifactorial; Nutritional deficiencies (folic acid, vitamin B12, etc.), genetic predisposition, and environmental factors play a role in the etiology [2, 7–9]. Geography, race, maternal obesity, antiepileptic drug use, pesticide exposure, smoking, indoor air pollution from predominantly biomass heating, x-ray radiation exposure, family history, and previous history of stillbirth have been reported to contribute to the development of NTDs [10–13]. Previous studies have shown that a significant proportion of NTDs can be prevented with pre-pregnancy folic acid supplementation (PFAS) [14–16]. In Turkey, PFAS has been recommended by health professionals for women of childbearing age and included in the Ministry of Health (MoH) guidelines since 2014 [17, 18]. Counselling and training services promoting PFAS are provided through the Premarital Counselling Program and pregnancy preparation classes in Maternity Schools [19].

Hospital-based studies conducted in previous years indicate that NTDs are common in Turkey [7, 11, 20–24]. However, there is no recent study evaluating NTD frequency with national data after the PFAS recommendation and there is no national study examining NTD mortality. Therefore, we designed this study to achieve the following objectives: a) To determine the frequency of patients with NTD and the NTD case fatality rate in a nationwide birth cohort in 2018, and b) To examine the epidemiological characteristics of NTD related deaths within the first 1 year of life using nationally collected data from 2014 to the end of 2019. The study is expected to be a reference study of preventive strategies.

## Methods

### Data sources

The study was designed as a descriptive study. We obtained the data through the following national data collection systems: a) The National Health Data System (e-Nabız), b) Death Notification System (DNS), c) Birth Notification System (BNS), d) Turkish Statistical Institute (TSI) birth data.

Based on WHO guideline and ICD-10 codes, NTD cases were examined in three subheadings: anencephaly and similar malformations (Q00.0; Q00.1; Q00.2), encephalocele (Q01.0; Q01.1; Q01.2; Q01.8; Q01.9) and spina bifida (Q05.0- Q05.9) [1]. In cases with more than one NTD anomaly, upper-level anomaly was considered as the main diagnosis.

### Stage I

The e-Nabız is personal health data recording and monitoring system of the MoH, Turkey that citizens and health workers can securely access health data collected from health institutions. In addition, this health information infrastructure enables the processing of collected data.

The 2018 birth cohort includes all babies born between January 1, 2018 and December 31, 2018, who have registered in the e-Nabız System.

In order to determine the frequency of NTD at birth, children diagnosed with NTD in the 2018 birth cohort were searched in the e-Nabız System using ICD-10 diagnostic codes.

In the list taken from this system, there were multiple applications of patients with the determined NTD ICD-10 codes, citizenship numbers, birth and death dates, the name of the health institution, the date of application and the interventions. Multiple applications of the patients were analyzed by the study group and singularized. The e-Nabız data was then combined with DNS data of the 2018 birth cohort including NTD related infant mortality, stillbirth, and ToPFA cases.

The combined list was deduplicated by the working group using maternal and/or infant citizenship numbers, personal BNS and DNS information. As a result, a list of NTD cases born (dead or alive) in 2018 was obtained through different data collection systems, with all cases unique. To determine the case fatality rate of NTD cases born in 2018 were tracked in DNS registries through their citizenship numbers until the end of March 2022.

### Stage II

Data on mortality were obtained from the DNS database. All infant deaths under 12 months (born alive at any gestational age and weight); ToPFA and stillbirth cases (more than 22 weeks of gestation or at least 500 g) are registered in DNS and reviewed by “Provincial Infant Mortality Monitoring Committees” and the main, intermediate and final causes are determined. All infant deaths, ToPFA and stillbirth cases registered in DNS between January 1, 2014 and December 31, 2019 were included in the study.

Among all deaths, the deaths having any of the NTD codes as their main or underlying cause of death were identified by the working group. A data sheet for evaluating each NTD mortality case was filled out with: type of mortality, ICD-10 codes, date of birth, date of death, gender, gestational age at birth, birth weight, maternal age, maternal and paternal education, household size, consanguineous marriage history, miscarriage/stillbirth history, maternal smoking, pregnancy type, parity, number of antenatal care, number of fetuses, delivery type, province of residence, forensic outcome, and autopsy result. The province of residence data was grouped according to five demographic regions of the country: West, South, Central, North, and East given in Demographic and Health Surveys [25].

The birth numbers referred to the relevant years in the calculation of cause-related death rates are taken from TSI. The numbers of births by socio-demographic and obstetric characteristics were obtained from the TSI and the BNS.

### Statistical analysis

We analyzed the data using Microsoft Office Excel 2019 and SPSS ver. 23.0 statistical software package. Arithmetic mean and standard deviation for continuous variables and frequency and percent distributions for categorical variables were used. For group comparisons of categorical and continuous variables, chi-square test and Student’s t-test were used respectively. When mortality rates with variables including year, region, and maternal age having more than two categories were found to be significant in Chi-square test, adjusted standardized residuals were calculated with Bonferroni method to detect subgroup differences.

Type I error was pre-set at 0.05 for all analyses.

### Ethical considerations

Hacettepe University, Non-Interventional Clinical Research Ethics Committee approved the study (2021/08–39). Official permission was obtained from the General Directorate of Public Health for sharing and analysis of the DNS data (official permission date and number: April 7th, 2021; 67,414,668–234.02-228). The survey was performed according to standards of the Declaration of Helsinki.

## Results

During 2014–2019 total of 7,736,309 live births, 53,504 stillbirths, and 4018 ToPFA cases were registered (Table 1). Of the live births, 73,698 resulted in infant deaths. While overall stillbirth rate decreased from 74.3 in 2014 to 64.0 in 2019 (per 10,000 live births and fetal deaths), overall infant mortality rate (IMR) decreased from 101.5 in 2014 to 90.3 in 2019 (per 10,000 live births). In contrast, overall ToPFA cases rose from 4.8 in 2014 to 6.4 in 2019 (per 10,000 live births) (Table 1).

**Table 1:** NTD related stillbirth, infant deaths and ToPFA cases, Turkey, 2014–2019

	2014–2019	2014	2015	2016	2017	2018	2019	Change 2014–2019, %
**Number of live births, N**	7,736,309	1,351,088	1,336,422	1,314,764	1,297,638	1,252,745	1,183,652	
**Number of infant deaths, N**	73,698	13,712	13,309	12,746	11,721	11,517	10,693	
**Number of stillbirths, N**	53,504	10,115	9490	9394	8746	8131	7628	
**Number of ToPFA, N**	4018	644	610	674	613	714	763	
**NTD related infant deaths, n (%)**^d^	2088 (2.8)	315 (2.3)^a^	315 (2.4)^a^	391 (3.1)^bc^	338 (2.9)^b^	390 (3.4)^c^	339 (3.2)^bc^	
**NTD related stillbirth, n (%)**^**d**^	1352 (2.5)	261 (2.6)	220 (2.3)	261 (2.8)	200 (2.3)	231 (2.8)	179 (2.3)	
**NTD related ToPFA, n (%)**^**d**^	471 (11.7)	70 (10.9)^a^	47 (7.7)^a^	57 (8.5)^a^	64 (10.4)^a^	112 (15.7)^b^	122 (16.0)^b^	
**IMR (per 10,000 live births)**	95.3	101.5	99.6	96.9	90.3	91.9	90.3	−11.0
**NTD specific IMR (per 10,000 live births)**	2.70	2.33^a^	2.36^a^	2.97^b^	2.60^ab^	3.11^b^	2.86^ab^	26.1
**Stillbirth rate (per 10,000 live births and stillbirths)**	68.7	74.3	70.5	70.9	66.9	64.5	64.0	−16.0
**NTD specific stillbirth rate** **(per 10,000 live births and stillbirths)**	1.74	1.92	1.65	1.97	1.53	1.83	1.50	−11.8
**TOPFA rate (per 10,000 live births)**	5.2	4.8	4.6	5.1	4.7	5.7	6.4	33.3
**NTD specific ToPFA rate (per 10,000 live births)**	0.61	0.52^a^	0.35^a^	0.43^a^	0.49^a^	0.89^b^	1.03^b^	100.0

*NTD* Neural tube defect, *ToPFA* Termination of pregnancy for fetal anomaly, *IMR* Infant mortality rate

^a,b,c^Different letters are significant in the same row, *p* < 0.05

^d^Proportion of NTD related death numbers to total numbers of specific deaths of relevant years

### NTD prevalence and mortality in 2018 birth cohort

**Fig. 1:**
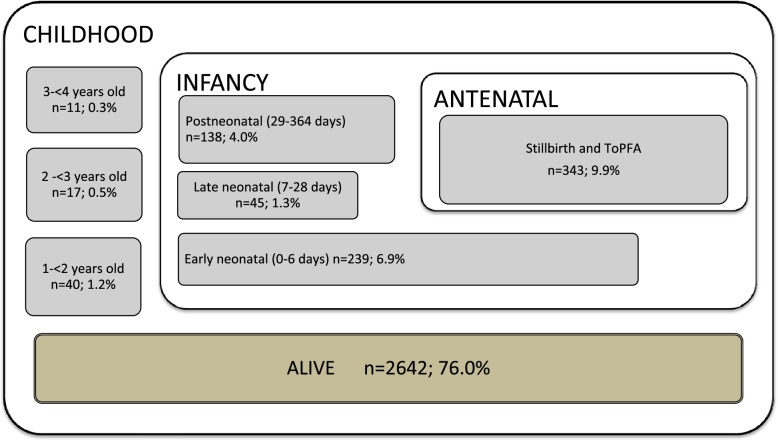
Distribution of fatality in cases diagnosed with NTD in the 2018 birth cohort by the end of March, 2022 (*n* = 3475)

In the 2018 birth cohort, analysis of multiple data systems showed that there were 3475 cases of NTD at birth (27.5 out of 10,000). Of the NTD cases, 9.9% (*n* = 343) were stillbirth and ToPFA cases, while 90.1% (*n* = 3132) were live-born infants with NTD. Of the NTD cases, 82.6% (*n* = 2869) were diagnosed with spina bifida, 12.2% (*n* = 425) with anencephaly, 4.9% (*n* = 169) with encephalocele, and 0.3% (*n* = 12) with unspecified NTD. The NTD cases in this cohort were tracked in DNS until the end of March 2022. The mortality age distribution is as follows: 6.9% in early neonatal, 1.3% in late neonatal, 4.0% in postneonatal, and 1.2% in 1- < 2 years-old, 0.5% in 2 - < 3 years-old, 0.3% in 3- < 4 years-old (Fig. 1). The case fatality rate of live-born babies with NTD in 2018 was 13.5% (422/3132) in the first year of life, and 15.6% (490/3132) at the end of March 2022.

### NTD-related ToPFA, stillbirth, and infant mortality cases (2014–2019)

NTDs were associated with 471 (11.7%) of ToPFA cases, 1352 of (2.5%) stillbirths, and 2088 (2.8%) of infant deaths over a 6-year period (2014–2019).

**Fig. 2:**
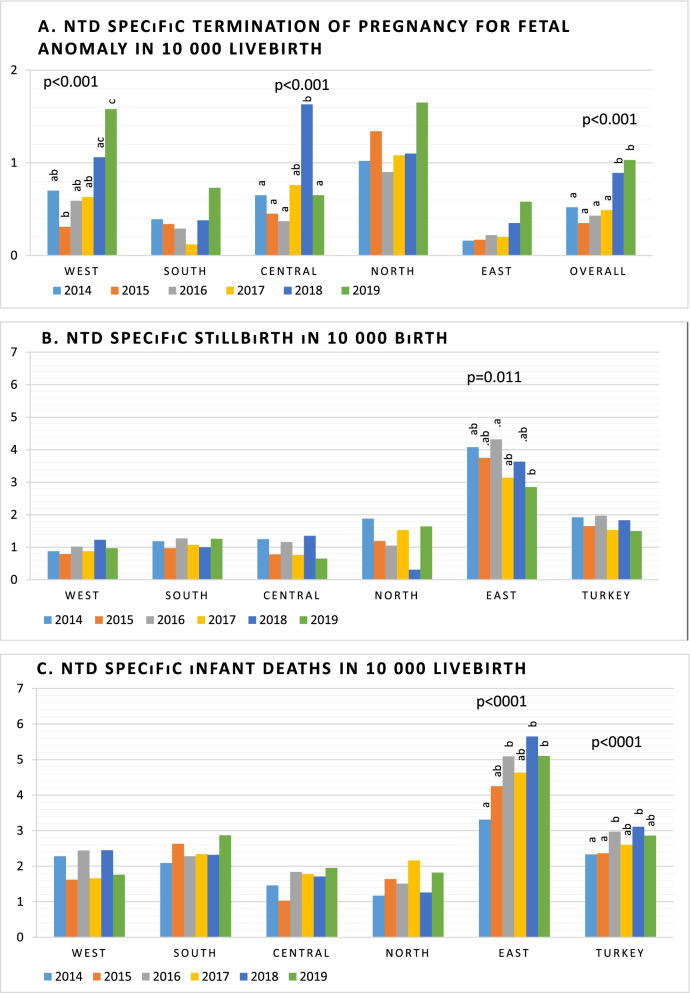
NTD specific mortalities (a. Termination of pregnancy for fetal anomaly; b. stillbirth; c. infant deaths) according to regions from 2014 to 2019

In overall, the NTD related stillbirth rate was 1.74 per 10,000 births (live births and stillbirths), while NTD related ToPFA was 0.61, and NTD related IMR was 2.70 per 10,000 live births. Table 1 shows that, while NTD related stillbirth rate exhibited decreasing trend (11.8% reduction), NTD related IMR exhibited an increasing trend (26.1%) between 2014 to 2019. NTD specific ToPFA rate has doubled in the same period (0.52 per 10,000 in 2014 to 1.03 per 10,000 in 2019) (Table 1). There are significant changes in NTD specific ToPFA rate and NTD specific IMR with time (*p* < 0.001 and *p* < 0.001, Fig. 2).

Among overall mortality cases, the most frequent category was anencephaly (55.3%), followed by spina bifida (32.6%), encephalocele (10.4%) and undefined NTD (1.8%). While in ToPFA cases, a greater proportion is due to spina bifida (53.5%), the vast majority of stillbirth cases are due to anencephaly (82.5%). In addition, proportion of undefined NTD group (8.9%) is higher in ToPFA cases than those of stillbirth (1.3%) and infant deaths (0.5%) (*p* < 0.001) (Table 2).

**Table 2:** Distribution of stillbirth, infant mortality and ToPFA by the type of NTD, Turkey, 2014–2019, (*n* = 3911)

NTD type	All NTD cases n (%)	NTD related ToPFA n (%)	NTD related stillbirths n (%)	NTD related infant deaths n (%)	NTD specific ToPFA (per 10, 000 live births)	NTD specific stillbirth rate (per 10, 000 live births and stillbirths)	NTD specific IMR (per 10,000 live births)
**Anencephaly**	2161 (55.3)	115 (24.4)^a^	1116 (82.5)^b^	930 (44.5)^c^	0.15	1.43	1.20
**Spina bifida**	1274 (32.6)	252 (53.5)^a^	161 (11.9)^b^	861 (41.2)^c^	0.33	0.21	1.11
**Encephalocele**	406 (10.4)	62 (13.2)^a^	57 (4.2)^b^	287 (13.7)^a^	0.08	0.07	0.37
**Undefined**	70 (1.8)	42 (8.9)^a^	18 (1.3)^b^	10 (0.5)^c^	0.05	0.02	0.01
**Total**	3911 (100.0)	471 (100.0)	1352 (100.0)	2088 (100.0)	0.61	1.74	2.70

*NTD* Neural tube defect, *ToPFA* Termination of pregnancy for fetal anomaly, *IMR* Infant mortality rate

^a,b,c^ Different letters are significant in the same row, *p* < 0.05

Table 3 shows the differences in NTD related stillbirths and infant deaths with ToPFA cases. Of all NTD related deaths, the male/female ratio was 1/1.7. Mean gestational age, mean birthweight, and mean maternal age were higher in stillbirth and infant deaths than ToPFA cases (*p* < 0.001; *p* < 0.001, and *p* = 0.033, respectively). Both maternal and paternal education were higher in ToPFA cases compared to stillbirth and infant deaths (*p* < 0.001 and *p* < 0.001, respectively). Household size was higher in stillbirth and infant deaths compared with ToPFA cases (*p* < 0.001). Higher nulliparity rate, higher single birth rate, and higher vaginal delivery rate were associated with ToPFA cases compared with stillbirth and infant deaths (*p* = 0.006, *p* < 0.001, and *p* < 0.001, respectively). While 51.0% of infant deaths and stillbirths occurred in the East Region, nearly half of the ToPFA cases (47.8%) were occurred in the West Region (Table 3).

**Table 3:** Differences in NTD-related stillbirth and infant mortality with late ToPFA cases, Turkey, 2014–2019, (*n* = 3911)

Variables	Overall	Stillbirth and infant death	ToPFA	p
**n**	**3911**	**3440**	**471**	
**Gender (%)**				0.160
Female	62.9	63.0	62.2	
Male	36.7	36.7	36.9	
Unknown	0.4	0.3	0.8	
**Gestational age (week)**	32.6 ± 5.8	33.7 ± 5.1	24.5 ± 3.4	< 0.001
**Birthweight (gr)**	1736 ± 996	1875 ± 971	731 ± 445	< 0.001
**Maternal age (year)**	28.5 ± 6.5	28.6 ± 6.5	27.9 ± 6.4	0.033
**Maternal education (year)**	5.6 ± 4.4	5.4 ± 4.3	8.1 ± 4.3	< 0.001
**Paternal education (year)**	6.9 ± 4.4	6.7 ± 4.4	8.5 ± 4.3	< 0.001
**Household size**	4.6 ± 2.7	4.7 ± 2.7	3.7 ± 1.9	< 0.001
**Consanguineous marriage (%)**				< 0.001
Yes	22.8	24.6^a^	9.8^b^	
No	71.4	69.5^a^	85.6^b^	
Unknown	5.8	5.9^a^	4.7^a^	
**Miscarriage/stillbirth history (%)**				0.002
Yes	30.1	31.0^a^	23.8^b^	
No	64.8	63.8^a^	72.0^b^	
Unknown	5.1	5.2^a^	4.2^a^	
**Maternal smoking (%)**				0.674
Yes	6.3	6.2	7.2	
No	85.6	85.6	85.1	
Unknown	8.1	8.1	7.6	
**Pregnancy type (%)**				0.118
Normal	94.1	93.8	96.2	
Assisted reproductive technology	2.2	2.3	1.3	
Unknown	3.7	3.9	2.5	
**Parity(%)**				0.006
Nullipar	24.2	23.4^a^	30.1^b^	
Multipar	72.1	72.8^a^	66.7^b^	
Unknown	3.7	3.7^a^	3.2^a^	
**Number of antenatal care**	5.9 ± 5.0	6.0 ± 5.1	5.7 ± 4.7	0.341
**Number of fetuses in the pregnancy (%)**				< 0.001
Singleton	91.9	91.3^a^	96.4^b^	
Twin/triplet	4.2	4.6^a^	1.1^b^	
Unknown	3.9	4.1^a^	2.5^a^	
**Delivery type (%)**				< 0.001
Vaginal delivery	44.3	39.4^a^	80.3^b^	
Cesarean section	50.8	55.7^a^	15.3^b^	
Unknown	4.9	5.0^a^	4.5^a^	
**Regions (%)**				< 0.001
West	27.5	24.7^a^	47.8^b^	
South	10.0	10.3^a^	7.9^a^	
Central	12.1	10.7^a^	22.3^b^	
North	4.0	3.3^a^	9.8^b^	
East	46.4	51.0^a^	12.3^b^	

*NTD* Neural tube defect, *ToPFA* Termination of pregnancy for fetal anomaly

Values are column percentage or mean ± SD

^a,b^Different letters in the same row are significant

### NTD related stillbirths and infant death rates by sociodemographic and obstetric characteristics

Table 4 shows the NTD specific stillbirth, infant death, and ToPFA rates by sociodemographic and obstetric characteristics. Higher NTD-related stillbirth, infant mortality and ToPFA rates were associated with multiparity, prematurity, female infant, and low birth weight. NTD-related stillbirth rates and ToPFA rates were lower in maternal age between 20 and 34 years than their counterparts. NTD specific stillbirth rates was detected to be highest in young mother and NTD specific infant death rates was highest in maternal age ≥ 35 yrs. Higher NTD specific infant death were found in twin/triplet foetuses. Caesarean delivery was associated with lower NTD specific stillbirth and ToPFA rates, and higher NTD specific infant death rates.

**Table 4:** NTD specific stilbirths, infant death, and ToPFA rates by sociodemographic and obstetric characteristics, Turkey, 2014–2019

Variables	Stillbirth rate^h^	NTD specific stillbirth N (rate)^h^	IMR^i^	NTD specific infant deaths N (rate)^i^	ToPFA rate^i^	NTD specific ToPFA N (rate)^i^
**Regions**^f^						
West	64.8^ab^	273 (0.96)^a^	77.4^a^	576 (2.04)^a^	7.3^a^	225 (0.79)^a^
South	66.4^a^	113 (1.12)^a^	93.4^b^	241 (2.41)^b^	4.2^b^	37 (0.37)^b^
Central	63.8^b^	142 (1.00)^a^	81.6^c^	228 (1.62)^c^	6.1^c^	105 (0.75)^a^
North	60.1^c^	50 (1.27)^a^	77.0^a^	62 (1.58)^ac^	9.6^d^	46 (1.17)^c^
East	79.7^d^	774 (3.64)^b^	132.5^d^	981 (4.65)^d^	1.4^e^	58 (0.27)^b^
**Maternal age**^f^						
< 20 yrs	65.2^a^	131 (3.04)^a^	106.6^a^	104 (2.43)^a^	4.0^a^	35 (0.82)^a^
20–34 yrs	6.2^b^	960 (1.58)^b^	87.9^b^	1534 (2.52)^a^	4.7^b^	349 (0.57)^b^
≥ 35 yrs	106.9^c^	260 (2.22)^c^	121.4^c^	442 (3.81)^b^	8.3^c^	87 (0.75)^a^
**Parity**^g^						
Nullipar	47.4^a^	45 (1.03)^a^	69.6^a^	60 (1.38)^a^	6.1	29 (0.67)^a^
Multipar	67.8^b^	130 (1.73)^b^	97.4^b^	276 (3.69)^b^	6.6	93 (1.24)^b^
**Gestational age**^g^						
≥ 37 mo	16.3^a^	25 (0.24)^a^	37.5^a^	157 (1.49)^a^	0.1^a^	1 (0.01)^a^
< 37 mo	431.0^b^	154 (11.26)^b^	512.2^b^	182 (13.91)^b^	57.3^b^	121 (9.25)^b^
**Number of fetuses**^g^						
Singleton	57.1^a^	169 (1.47)	76.6^a^	311 (2.72)^a^	6.3	121 (1.06)
Twin/triplet	150.8^b^	6 (1.51)	408.3^b^	28 (7.17)^b^	7.7	1 (0.26)
**Delivery type**^g^						
Vaginal	78.9^a^	106 (2.10)^a^	67.1^a^	69 (1.38)^a^	12.3^a^	97 (1.94)^a^
Cesarean section	46.6^b^	69 (1.01)^b^	102.1^b^	266 (3.89)^b^	2.2^b^	25 (0.37)^b^
**Gender**^f^						
Male	68.3	444 (1.11)^a^	101.1^a^	818 (2.06)^a^	4.9	174 (0.44)^a^
Female	68.2	898 (2.37)^b^	88.6^b^	1270 (3.37)^b^	5.1	293 (0.78)^b^
**Birth weight**^g^						
≥ 2500 g	18.2^a^	10 (0.09)^a^	36.8^a^	148 (1.36)^a^	1.4^a^	1 (0.01)^a^
< 2500 g	557.5^b^	169 (16.80)^b^	698.8^b^	191 (20.11)^b^	20.0^b^	121 (12.74)^b^

*NTD* Neural tube defect, *ToPFA* Termination of pregnancy for fetal anomaly

^a,b,c,d,e^Different letters in the same column are significant for each variable, *p* < 0.05

^f^2014–2019 data

^g^Only 2019 data

^h^in 10,000 birth

^i^in 10,000 livebirth

When regional differences were examined, it was found that NTD related stillbirth rate and NTD related IMR were found to be significantly higher in the East region (3.63 per 10,000 birth and 4.65 per 10,000 livebirth, respectively) compared to other regions (*p* < 0.05). In contrast, NTD related ToPFA rate was higher in the North (1.17 per 10,000 livebirth) compared to other regions, while it was lowest in the South region (0.37 per 10,000 livebirth) and the East region (0.27 per 10,000 livebirth) (*p* < 0.05) (Table 4). NTD related ToPFA rate were detected to be change with time in the West and the Central region. NTD related IMR was found to be increased in the East from 2014 to 2019 (*p* < 0.001, Fig. 2). Compared to 2019, NTD related stillbirth rate was higher in 2016 (*p* < 0.05).

## Discussion

In the current study, NTDs were evaluated using available national data and the results showed that NTDs are still prevalent (27.5 per 10,000 births) in Turkey. In previous years, the epidemiology of NTDs have been studied in several studies, with varying results [7, 11, 20–22, 24]. According to the results of studies conducted in various provinces before the 2000s, the frequency of NTD was reported to vary between 30 and 58 per 10,000 births [11, 20, 21, 23]. In a large-scale, nationally representative multicenter study of 21,907 children born between June 1993 and July 1994 in university clinics, the frequency of NTD was found to be 30.1 per 10,000 births [11]. A regional nested case-control study in İzmir province detected the incidence as 15.0 per 10,000 births in 2000 [24]. Another study evaluating all live births, stillbirths and therapeutic abortions, based on records of hospitals in Afyonkarahisar province in 2003–2004 reported the incidence of NTD as 35.8/10000 births [22]. The NTD rate in a tertiary care referral perinatology unit form low-income region of Eastern Turkey between 2016 and 2018 was 130.0 (151/11552) per 10,000 fetuses [7]. Differences in the results of these studies may be due to differences in time, geography, and population, as well as methodological differences such as data collection criteria, case definition, and study design. In a study estimating the burden of NTD in low- and middle-income countries showed that total NTD burden was 25.5 (IQR 15.6, 39.1) per 10,000 pregnancies [26]. A systematic review including 160 full text manuscripts and reports from 75 countries demonstrated that prevalence estimates vary widely by region: Africa; 11.70 per 10,000 birth (95% CI 5.20, 75.40); Eastern Mediterranean: 21.90 per 10,000 birth (95% CI 2.10, 124.10); European: 9.00 per 10,000 birth; (95% CI 1.30, 35.90), Americas: 11.50 per 10.000 birth (95% CI 3.30, 27.90), South-East Asia: 15.80 per 10,000 birth (95% CI 1.90, 66.20), and Western Pacific: 6.90 per 10,000 birth (95% CI 0.30, 199.40) [27]. In a meta-analysis study that included 19 studies from 1968 to 2013, the overall pooled prevalence of NTDs in India was 45.00 per 10,000 total births (95% CI 42.00, 49.00) [28]. In a recent study, which included a meta-analysis of 37 publications from the African region in 1990 and 2020, the prevalence of NTDs was reported as 50.71 (95% CI 48.03, 53.44) per 10,000 births [10].

NTD is a congenital anomaly with high mortality and morbidity. In the current study, the case fatality rate in live-born babies was 13.5% in the first year of life, and 15.6% at the end of March 2022. In a study from a tertiary care center in Konya, analyzing patients followed in with a diagnosis of NTD, the case fatality rate during the follow-up period was 7.5%. In another study from Samsun, analyzing patients followed in the neonatal intensive care unit with a diagnosis of NTD, the mortality rate during the follow-up period was 7.0% [29]. Since our study reflects the results of the whole country and all NTD types including anencephaly, higher mortality is expected compared to results from this well-equipped tertiary referral centre. In a systematic review conducted in high-income country settings including twenty studies, significant declines in spina bifida associated IMR (4.8% decrease in IMR per 100,000 live births) and case fatality (2.7% decrease in infant case fatality) were reported [30]. Possible reasons for this difference are reported as: primary prevention with mandatory folic acid fortification, early prenatal diagnosis with improved technology, termination of pregnancy, and advances in therapeutic medical and surgical interventions including improved ventilator support in neonatal intensive care and the use of antibiotics [30].

Our study showed that NTDs account for a significant proportion of infant deaths, stillbirths, and ToPFA. While overall IMR and stillbirth rate tend to decline, NTD-related IMR and ToPFA rates tend to increase over the years. NTD-specific stillbirth rates follow an irregular course. Although this result may seem contradictory, it was interpreted as a gradual improvement in the quality of the data entered on causes of death. There are also regional differences in NTD specific IMR, stillbirth and ToPFA rates. NTD-associated IMR and stillbirth are the highest, while NTD related ToPFA is the lowest in the Eastern region when compared to other regions. In the recently published report by the MoH, it was shown that both IMR and perinatal mortality rates are higher in the Eastern region, in line with our study results [31, 32]. In this study, we calculated NTD related stillbirths, ToPFA and infant death rates by some socio-demographic and obstetric characteristics. Higher NTD-related infant mortality rates were associated with low birth weight, prematurity, old maternal age, twin/triplet pregnancy, multiparity, and cesarean delivery whereas NTD-related stillbirth and ToPFA rates was higher in: low birth weight, prematurity, young maternal age, female infant, vaginal delivery, and multiparity. The highest rates for NTD specific mortality were in the prematurity and low birthweight variables. In a new meta-analysis study, similar to our study, prematurity and low birthweight are reported to be associated with increasing spina bifida infant case fatality [30].

In the current study, one out of 8 to 9 cases of all ToPFA cases were found to have a diagnosis of NTD. We showed that some socio-demographic characteristics of NTD related ToPFA cases differ from NTD related infant deaths and stillbirths. In ToPFA cases; mothers were younger, nulliparous, and had less history of miscarriage/stillbirth, parents were more educated, the house was less crowded, twin/triplet pregnancies and consanguineous marriages were less than those other death types. The regional distribution of NTD related ToPFA cases were also different. While 51.0% of stillbirth and infant death cases are seen in the Eastern region, 47.8% of NTD related ToPFA cases occurred in the Western region. However, the highest ToPFA rates were found in the Northern region and Western region respectively. In Turkey, abortion is legally allowed until the tenth week of pregnancy is completed. Termination of pregnancy due to serious diseases is done after the decision of the health board, regardless of the week of gestation [33]. In a study assessing ToPFA cases performed before the 16th week of gestation, cranial NTD and spina bifida accounted for 35.9% of all cases [34]. As a limitation of the current study, this study included only late ToPFA cases. While the gestational age at termination of pregnancy could be taken from the records, the gestational age at the diagnosis could not be obtained. Further studies that include both qualitative and quantitative components are needed to better analyze NTD-related termination cases.

Although the evidence is strong enough that PFAS can prevent a significant proportion of NTDs, it has been shown that the use of individual supplements is not effective enough to prevent NTD at the population level. Because unplanned pregnancies are common, and pre-pregnancy health check-up and use of folic acid supplementation before conception require a high level of awareness [35]. For this reason, mandatory folic acid fortification programs have launched to be implemented and a significant reduction in the prevalence of NTDs has been reported following mandatory fortification with folic acid in many countries [36–40]. Long term surveillance data from the countries showed that the NTD birth prevalence can be reduced over time to as low as 5–6 per 100,000 pregnancies [9, 16, 41, 42]. However, legislative regulations mandating the fortification of staple foods with folic acid lag behind evidences, especially in Asian and European countries [43, 44]. Currently, in Turkey, a program regarding fortifying staple foods with folic acid is not implemented. Furthermore, according to Turkish Nutritional Guide-2015, > 30% of women of childbearing age were found to have folate intake below the estimated average requirement [45]. A recent study determined that 40.2% of women over the age of 15 had a folate deficiency or insufficiency (< 5.9 ng/mL) [46]. The results of our study and these supporting findings indicate the need for a public health intervention for the prevention of NTDs in Turkey.

### Strengths and limitations

This study had several strengths. The study included 6 years of national mortality data, which allowed assessment of nationwide trends. Our results show the actual status in the community. Furthermore, we analyzed not only infant mortality, but also stillbirth and ToPFA cases. But, as a limitation of the study, cases < 22 GH or < 500 g (miscarriages and early medical terminations of pregnancy) could not be included. For this reason, the total number of pregnancies and fetuses affected by NTD could not be detected. Another major limitation of the study was the secondary analysis nature, so all the independent variables examined were limited to those found in the database. The causes of death entered into the database and use of appropriate disease-specific ICD-10 codes are directly related to the knowledge and awareness of health professionals.

## Conclusions

The results of this study revealed that, despite the PFAS recommendation and promotion, NTD is still common in our country and has an important place among the causes of death. At this stage, primary prevention through mandatory folic acid supplementation seems to be the more rational solution in line with global progress. Initiation of a public health program to prevent NTD cases by fortifying staple foods with folic acid should be urgently addressed. A congenital anomaly surveillance system establishment should also be considered to monitor the progress.

## Data Availability

The data of this study are available from the Ministry of Health.
